# Photodynamic diagnosis of shed prostate cancer cells in voided urine treated with 5-aminolevulinic acid

**DOI:** 10.1186/1471-2490-14-59

**Published:** 2014-08-03

**Authors:** Yasushi Nakai, Satoshi Anai, Masaomi Kuwada, Makito Miyake, Yoshitomo Chihara, Nobumichi Tanaka, Akihide Hirayama, Katsunori Yoshida, Yoshihiko Hirao, Kiyohide Fujimoto

**Affiliations:** 1 Department of Urology, Nara Medical University, 840 Shijo-cho, Kashihara-shi, Nara 634-8522, Japan

**Keywords:** 5-aminolevulinic acid, Prostate cancer, Photodynamic diagnosis, Urine cytology

## Abstract

**Background:**

Past attempts at detecting prostate cancer (PCa) cells in voided urine by traditional cytology have been impeded by undesirably low sensitivities but high specificities. To improve the sensitivities, we evaluate the feasibility and clinical utility of photodynamic diagnosis (PDD) of prostate cancer by using 5-aminolevulinic acid (5-ALA) to examine shed prostate cancer cells in voided urine samples.

**Methods:**

One hundred thirty-eight patients with an abnormal digital rectal exam (DRE) and/or abnormal prostate-specific antigen (PSA) levels were recruited between April 2009 and December 2010. Voided urine specimens were collected before prostate biopsy. Urine specimens were treated with 5-ALA and imaged by fluorescence microscopy and reported as protoporphyrin IX (PPIX) positive (presence of cells demonstrating simultaneous PPIX fluorescence) or PPIX negative (lack of cells demonstrating fluorescence).

**Results:**

Of the 138 patients, PCa was detected on needle biopsy in 81 patients (58.7%); of these 81 patients with PCa, 60 were PPIX-positive (sensitivity: 74.1%). Although 57 patients did not harbor PCa by conventional diagnostic procedures, 17 of these at-risk patients were found to be PPIX-positive (specificity: 70.2%). PPIX–PDD was more sensitive compared with DRE and transrectal ultrasound and more specific compared with PSA and PSA density. The incidence of PPIX–PDD positivity did not increase with increasing total PSA levels, tumor stage or Gleason score.

**Conclusions:**

To our knowledge, this is the first successful demonstration of PPIX in urine sediments treated with 5-ALA used to detect PCa in a noninvasive yet highly sensitive manner. However, further studies are warranted to determine the role of PPIX–PPD for PCa detection.

## Background

Detection of prostate cancer (PCa) primarily relies on an abnormal digital rectal examination (DRE) and/or increased prostate specific antigen (PSA) levels. Despite its widespread use, PSA has marginal specificity, and 65%–70% males with elevated PSA levels within 4–10 ng/ml generally reveal a negative biopsy result [1]. Therefore, PSA is not the ideal screening tool for prostate cancer. To improve PSA specificity, various analyses have been introduced such as PSA density, PSA velocity, and free/total PSA; however, these methods have not appreciably improved the cancer detection rates. In addition, because of sampling errors or sampling inefficiencies associated with transrectal ultrasound-guided biopsy of the prostate, some PCa may be missed (false negative). Therefore, there is a clear need for clinically useful biomarkers that would allow earlier detection of clinically significant PCa. For this reason, there is currently considerable interest in novel noninvasive biomarkers (e.g., urine- or serum-based assays) to assist in the diagnosis of PCa [2]. Nevertheless, to date, no routinely used tumor biomarker has replaced PSA.

Upon prostatic massage, prostate cells (including PCa cells) may be dislodged and displaced into the urethra and urine. However, unlike bladder cancer, wherein voided urinary cytology has proven useful in cancer detection and surveillance, past attempts at detecting PCa cells in voided urine by traditional cytology have been impeded by undesirably low sensitivities but high specificities [3-5]. The low sensitivity was presumably attributed to the scant number of prostate cells present in the voided urine and the difficulty in differentiating the malignant prostatic cells from the other shed cells and debris. Thus, based on recent technological advancements, we revisited the cytology-based approach to PCa detection in voided urine samples and investigated the use of photodynamic diagnosis (PDD). For example, in bladder cancer, several investigators have used photodynamic agents such as 5-aminolevulinic acid (5-ALA) to induce protoporphyrin IX (PPIX) accumulation in malignant tissue, which then enables it to be differentiated from benign tissue [6-9]. In the heme biosynthetic pathway, 5-ALA is a precursor in the heme biosynthesis pathway and is metabolized to fluorescent PPIX before being converted to photoinactive heme. PPIX, which temporarily accumulates in the cancer cells after exogenous application of 5-ALA, is the endogenous photosensitizer needed for PDD [10]. Selective PPIX accumulation in malignant tissue provides an intense color contrast between the red fluorescence of malignant lesions and the nonfluorescence of normal tissue.

Previously, Zaak et al. reported the results of a small feasibility study (n = 18), in which prostate tissue samples were analyzed after exposure to 5-ALA for the presence of prostate tumor(s). Briefly, prostate tissue samples from 16 patients who had undergone 5-ALA fluorescence microscopy revealed selective PPIX accumulation in the PCa cells, and only weak PPIX fluorescence could be detected in the benign epithelial cells. The two patients who were not treated with 5-ALA revealed no PPIX fluorescence within the prostate [11]. In this study, our aim was different from those of previous investigators. We evaluated the feasibility and clinical utility of PDD for PCa by using 5-ALA-induced PPIX to examine shed PCa cells in voided urine samples. The use of a fluorescence biomarker for the diagnosis of cancers is an intriguing and still fledgling concept for improving the detection accuracy of PCa.

## Methods

### Patients

After receiving approval from the Nara Medical University Hospital Institutional Review Board, 138 patients with an abnormal DRE and/or abnormal PSA levels were recruited between April 2009 and December 2010. All patients provided written informed consent and were subsequently enrolled in this prospective feasibility study. Patients with a past history of urothelial carcinoma were not eligible for this study. For all the patients, before transrectal ultrasound needle-guided biopsy of the prostate, an “attentive” (approximately 30 s) DRE was performed, and the first 50 ml of voided urine was collected. Thereafter, the patients underwent prostate needle biopsies at Nara Medical University Hospital. Biopsies were performed by adjusting the number of cores sampled (6–12) according to the age of the patient and the prostatic volume. The medical records of our patients were reviewed for demographics, clinical, and pathological information (e.g., tumor grade, Gleason score, and tumor stage) as well as outcome. The clinicopathological characteristics of this cohort are listed in Table 1. All voided urine specimens were stored at 4°C until processed, which typically occurred within 2 h of collection.

**Table 1:** Characteristic of the study population

	**Males with positive biopsy (n = 81)**	**Males with negative biopsy (n = 57)**		**Total (n = 138)**
	**Median (range)**	**Median (range)**	**p-value**	**Median (range)**
Age (years)	72 (51-86)	67 (55-81)	0.002	70 (51-86)
PSA (ng/ml)	9.7 (3.8-690)	7.4 (1.4-24.1)	0.002	8.3 (1.4-690)
Free PSA (ng/ml)	1.5 (0.46-95)	1.2 (0.15-3.3)	0.008	1.4 (0.15-95)
%fPSA (%)	15 (3-38)	17 (2-39)	0.679	15 (3-39)
PV (cm^3^)	28.7 (11.5-95.2)	34.0 (16.2-111)	0.002	30.4 (11.5-111)
TZV (cm^3^)	13.3 (3.7-45)	16.9 (2.9-78)	0.04	15.2 (2.9-78)
PSAD (ng/ml/cm^3^)	0.39 (0.07-46.6)	0.20 (0.06-1.0)	<0.001	0.27 (0.06-46.6)

*Abbreviation:SD* standard deviation, *PSA* prostate-specific antigen, *%fPSA* percent-free PSA, *PV* prostate volume, *TZV* transition zone volume, *PSAD* PSA density.

### Sample preparations and 5-ALA treatment

**Figure 1:**
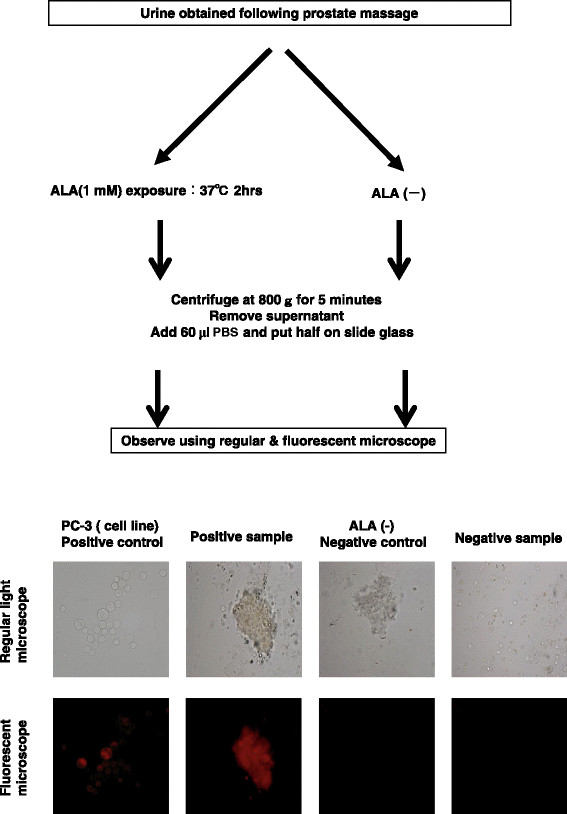
**Workflow of the procedure and representative PPIX–PDD staining.** DRE, digital rectal examination; ALA, aminolevulinic acid; RPM, revolutions per minute; PBS, phosphate buffered saline.

Urine specimens were centrifuged at 800 g for 5 min. The supernatant was decanted, and the pellets were suspended in phosphate-buffered saline (PBS) buffer, from which two samples were collected. One sample was treated with 1-mM 5-ALA (Sigma-Aldrich Co. Saint Louis, MO, USA), diluted with PBS, and then incubated at 37°C for 2 h. The solution was centrifuged at 5000 *g* for 5 min, the supernatant was decanted, and 60 μl of PBS was added to suspend the cells. Next, the cells were applied to a charged-surface microscope slide (Figure 1). The second aliquot of shed cells within the urine was similarly processed, except that it was not treated with 5-ALA. Furthermore, a minute amount of material was removed from the second aliquot in which RNA was extracted from the cells by using the RNeasy Mini kit (Qiagen, Hilden, Germany) as per the manufacturer’s instructions. A High-Capacity cDNA Reverse Transcription kit (Life Technologies, Palo Alto, CA) was used for the conversion cDNA. Primer sets for PSA (fp- 5′ TGACCAAGTTCATGCTGTGT3′ and rp- 5′ TCCTTGGAGGCCATGTGGGCCAT 3′) were used to perform quantitative reverse transcription polymerase chain reaction (RT-PCR). Further, the relative fold changes in mRNA levels were calculated after normalization to glyceraldehyde 3-phosphate dehydrogenase (GAPDH).

### Photodynamic detection of PPIX in shed prostate cells

5-ALA-treated (and 5-ALA-untreated) urine cytospin slides were investigated for PPIX fluorescence at 100× magnification by fluorescent microscopy with the appropriate fluorescence filter sets (excitation filter, 380–420 nm; emission filter, 590 nm; Nikon Eclipse 400; Nikon Instruments Inc., Melville, NY, USA). The slides were evaluated for the following: (i) PPIX positivity, presence of cells demonstrating simultaneous fluorescence of PPIX and (ii) PPIX negativity, lack of cells demonstrating fluorescence.

### Statistical analysis

The Mann–Whitney test was used to analyze the differences between the patients with or without PCa. We used the Chi-square test for trend to analyze whether the incidence of PPIX–PDD positivity was associated with cancer, PSA, Gleason score, and tumor stage. Multivariate logistic regression analysis was used to evaluate the demographic features, key clinical features [e.g., DRE, total PSA,%fPSA, PSA density (PSAD), and transrectal ultrasonography (TRUS)], and the PPIX–PDD results in order to determine their ability to independently predict PCa on prostate biopsy. Differences were considered statistically significant at *p* values <0.05.

## Results

In this study, 81/138 (58.7%) patients revealed PCa on needle biopsy (Table 1). The median age of the patients without PCa was 67 (range 55–81) years, and the median age of patients with PCa was 72 (range 51–86) years (*p* = 0.002). The median PSA of the patients without PCa was 7.4 (range 1.4-24.1) ng/ml, and the median PSA of the patients with PCa was 9.7 (range 3.8-690) ng/ml (*p* = 0.002). Furthermore, the median free PSA (1.5 vs. 1.2 ng/ml, *p* = 0.008) and PSAD (0.39 vs. 0.20 ng/ml/cm^3^, *p* < 0.001) values differed significantly between the patients with and without PCa, respectively. In addition, prostate volume (34.0 *vs.* 28.7 ml, *p* = 0.002) and transition zone volume (16.9 *vs.* 13.3 ml, *p* = 0.04) were significantly higher in the patients with PCa than in those without PCa.

The typical fluorescence of urine sediments placed on a cytospin slide from a patient with PCa was compared to its corresponding unstained urine sediments (Figure 1). These cells were found to express PSA by RT-PCR and were thus considered to be prostate cells (data not shown). The human prostate cancer cell line PC-3 was used as a positive control. Table 2 compares the PPIX–PDD results with the other clinical parameters (e.g., DRE, PSA,%fPSA, PSAD, and prostate volume) associated with PCa detection. Of the 81 patients with biopsy-proven PCa, 60 were determined positive for PPIX–PDD (sensitivity: 74.1%). PPIX–PDD was more sensitive compared with DRE and TRUS and more specific compared with PSA and PSAD. Although 57 patients did not harbor PCa by conventional diagnostic procedures, 17 of these at-risk patients were determined positive for PPIX–PDD (specificity: 70.2%). These patients have been under close surveillance to evaluate if their positive PPIX–PDD results will convert to clinical PCa.

**Table 2:** Sensitivity and specificity of ALA, DRE, PSA, %sPSA, PSAD and TRUS

	**Sensitivity**	**Specificity**
ALA	74.1%	70.2%
DRE	44.4%	91.2%
PSAD (cut-off 0.15)	93.8%	29.8%
%fPSA (cut-off 20%)	78.7%	26.3%
PSA (cut-off 4ng/ml)	60.1%	50.0%
TRUS	34.6%	96.5%

*Abbreviation:ALA* aminolevulinic acid, *DRE* digital rectal examination, *TRUS* transrectal ultrasinography, *PSAD* PSA density, *%fPSA* percent free PSA.

The diagnostic accuracy of PPIX–PDD in the males with positive biopsy at different total PSA levels was 2/2 (100%) patients for PSA levels within 0–4 ng/ml, 28/39 (71.8%) for PSA levels within 4–10 ng/ml, 11/15 (73.3%) for PSA levels within 10–20 ng/ml, and 19/25 (76.0%) for PSA levels >20 ng/ml. The diagnostic accuracy at different tumor stages was 34/45 (75.6%) for tumor stage cT1, 22/32 (68.8%) for cT2, and 4/4 (100%) for cT3. The incidence of PPIX–PDD positivity did not increase with increase in the total PSA levels (*p =* 0.25) and tumor stage (*p =* 0.87).Furthermore, the diagnostic accuracy of PPIX–PDD in the males with positive biopsies was 15/24 (62.5%) for Gleason score (GS) 6, 26/34 (76.4%) for GS 7, and 19/23 (82.6%) for GS 8–10.The incidence of PPIX–PDD positivity increased with increasing GS, but the increase was not significant (*p =* 0.11) (Table 3).

**Table 3:** Diagnostic accuracy of PDD according to total PSA, grade (Gleason) and stage

	**Males with positive biopsy (n=81)**	**Males with positive PDD (n=60)**	**Sensitivity**	p-value*
0-4	2	2	100%	
4-10	39	28	71.8%	
10-20	15	11	73.3%	
20-	25	19	76.0%	0.25
Gleason score				
6	24	15	62.5%	
7	34	26	76.4%	
8-10	23	19	82.6	0.11
Clinical stage				
cT1	45	34	75.6%	
cT2	32	22	68.8%	
cT3	4	4	100%	
cT4	-	-	-	0.87

*Chi-square test for trend.

In addition, on multivariate analysis, we evaluated the demographic features, key clinical features (e.g., DRE, total PSA,%fPSA, PSAD, and TRUS), and PPIX–PDD results to determine their ability to independently predict PCa on prostate biopsy (Table 4). Multivariate analysis indicated that ALA (HR, 8.00; 95% CI, 2.50–25.59; *p* < 0.001) and PSAD (HR, 28.43; 95% CI, 8.12–99.58; *p* < 0.001) were independent diagnostic factors for PCa.

**Table 4:** Univariate and multivariate analysis of factors to predict PCa on prostate biopsy

	**Univariate analysis**			**Multivariate analysis**		
	**OR**	**95% CI**	**p-value**	**OR**	**95% CI**	**p-value**
ALA (positive/negative)	6.72	3.16-14.29	<0.001	8.00	2.50-25.59	<0.001
DRE (abnormal/normal)	8.00	2.89-22.15	<0.001	1.77	0.32-9.38	0.52
PSAD (>0.15/<0.15)	34.20	11.63-100.54	<0.001	28.43	8.12-99.58	<0.001
% fPSA (>20%/<20%)	1.57	0.71-3.53	0.27			
PSA (>20/>10, <20/<10)	2.37	1.43-3.95	0.001	1.97	0.81-4.80	0.14
TRUS (abnormal/normal)	13.50	3.06-59.66	0.001	7.35	0.52-104.58	0.14

*Abbreviation:OR* odds ratio, *ALA* aminolevulinic acid, *DRE* digital rectal examination, *PSAD* PSA density, *%fPSA* percent free PSA, *TRUS* transrectal uktrasonography.

## Discussion

In this study, we demonstrated the feasibility of PPIX–PDD and 5- ALA in noninvasive detection of PCa cells extracorporeally in voided urine sediments. The use of voided urine samples in an attempt to diagnosis PCa is gaining widespread attention. For example, investigators are using RT-PCR to identify the prostate cancer antigen 3 (PCA-3) [12] and glutathione S-transferase P1^12^ gene [13] in voided urine samples. Particularly, the sensitivity and specificity of PCA-3 have been reported to be 54% and 74%, respectively, for the entire cohort and 53% and 71%, respectively, for PSA within 4–10 ng/ml. In the present study, the sensitivity and specificity of PPIX–PDD were 74.1% and 70.2% in the entire cohort and 70.0% and 67.6% in the gray zone cases, which were superior to those reported for PCA-3. Interestingly, the positivity rate of PPIX–PDD did not increase with increasing total PSA or tumor stage. However, the positivity rate of PPIX–PDD did increase with increasing Gleason score though a non-significant trend (*p =* 0.11) was noted. These results showed a possibility that PCa detection with PPIX–PDD identified more clinically significant PCa compared with PCa detected by other reported methods. Stummer et al*.* reported that the fluorescence intensity of glioma by PPIX–PDD was associated with cellular density, tumor proliferation, and tumor angiogenesis [14]; these results supported our results indicating that PPIX–PDD detected more aggressive clinically relevant cancers.

Although no reports regarding fluorescent cytology of shed PCa cells were found in the literature, several reports from the bladder cancer literature are available [15-17]; in these reports, voided urine after intravesical 5-ALA instillation was evaluated. Inoue et al. reported that regardless of the 5-ALA administration route (intravesical or oral), exposure to 5-ALA was well tolerated with only cystitis symptoms being reported [18]. Hence, in this extracorporeal model, shed prostate cells rather than patients were treated with 5-ALA.

Although the findings in the present study were quite compelling, the study had some limitations. First, we obtained limited pathological data on the patients with positive PPIX–PDD results and positive prostate biopsy results who proceeded to undergo radical prostatectomy. Second, with limited follow-up (mean follow-up, 27 months), we must cautiously interpret the false-positive PPIX–PDD results. It is possible that over time, these prostate biopsies will become positive. In this cohort, only 4 false positive patients had been performed repeated prostate needle biopsies after this study. 2 patients of them were diagnosed as prostate cancer. These false positive patients may need strict PSA follow-up or a saturation biopsy. Third, we didn’t perform RT-PCR for every patient. We demonstrated RT-PCR in the first twenty patients. Then we evaluated the expression of PSA. We could confirm the existence of cells from prostate by that. In addition, these false-positive results may be attributed to autofluorescence. Tauber et al. reported that some biological materials had autofluorescence. To address this issue, the authors used bleaching fluorescence; however, the specificity of bleaching fluorescence was comparatively low [16]. In our study, we were able to subjectively estimate autofluorescence by examining the slide not treated with 5-ALA from each patient. Furthermore, cytological examinations are known to present interobserver variability. In the future, we hope to develop a more objective spectrophotometric method to quantify the intensity of PPIX fluorescence.

## Conclusions

To our knowledge, this is the first successful demonstration of PPIX in urine sediments treated with 5-ALA for detection of PCa in a noninvasive yet highly sensitive manner. The incidence of PPIX–PDD positivity increased with increasing Gleason score though a non-siginificant trend (*p =* 0.11) was noted. Further studies are warranted to determine the role of PPIX–PPD for PCa detection.

## Abbreviations

ALA: Aminolevulinic acid; DRE: Digital rectal exam; GAPDH: Glyceraldehyde 3-phosphate dehydrogenase; GS: Gleason score; PBS: Phosphate-buffered saline; PDD: Photodynamic diagnosis; PCa: Prostate cancer; PCA-3: Prostate cancer antigen 3; PSA: Prostate-specific antigen; PSAD: PSA density; PPIX: Protoporphyrin IX; TRUS: Transrectal ultrasonography; RT-PCR: Reverse transcription polymerase chain reaction.

## Competing interests

The authors declare that they have no competing interests.

## Authors’ contributions

YN and SA carried out the photodynamic detection and drafted the manuscript. SA also designed the experiments and interpreted the data. MK and MM helped with photodynamic detection and RT-PCR. YC provided valuable help with the experiments. NT contributed to the design and conception. AH revised this manuscript. KY and KF participated in study design. YH conceived of the study and participated in its design. All authors read and approved the final manuscript.

## Pre-publication history

The pre-publication history for this paper can be accessed here:

http://www.biomedcentral.com/1471-2490/14/59/prepub
